# Cleaning Methods for Ceramic Ultrafiltration Membranes Affected by Organic Fouling

**DOI:** 10.3390/membranes11020131

**Published:** 2021-02-14

**Authors:** Kamila Gruskevica, Linda Mezule

**Affiliations:** Water Research and Environmental Biotechnology Laboratory, Faculty of Civil Engineering, Riga Technical University, Paula Valdena 1-204/205, Riga LV-1048, Latvia; kamila.gruskevica@rtu.lv

**Keywords:** ceramic membranes, organic fouling, cleaning

## Abstract

The use of ceramic membranes in the treatment and processing of various liquids, including those of organic origin, has increased tremendously at the industrial level. Apart from the selection of the most appropriate membrane materials and operational conditions, suitable membrane cleaning procedures are a must to minimize fouling and increase membrane lifespan. The review summarizes currently available and practiced non-reagent and cleaning-in-place methods for ceramic membranes that are used in the treatment of organic liquids, thus causing organic fouling. Backflushing, backwashing, and ultrasound represent the most often used physical methods for reversible fouling treatment. At the same time, the use of alkalis, e.g, sodium hydroxide, acids, or strong oxidants are recommended for cleaning of irreversible fouling treatment.

## 1. Introduction

Ceramic ultrafiltration membranes are widely used in bioenergy, pharmaceutical and food production, drinking water and wastewater treatment, metal, coating, and textile industries. Most often, the membranes are made of Al, Si, Ti, and Zr oxides [1,2,3]. During production, oxides are sintered to a ceramic support layer to form a microporous inorganic top layer [4] with a typical configuration of flat-sheet, hollow fiber, or tubular. A three-layer structure with different porosity provides endurance, minimizes surface roughness, and ensures effective separation [1,2]. If compared to polymeric membranes, ceramic membranes can tolerate intense backflushing, harsh chemical treatments, and high temperatures up to 300 °C, work in an all-pH spectrum, and have a lifespan of 10–20 years [1,5,6,7,8]. Furthermore, ceramic membranes have higher porosity and more uniform pores [9], they are less susceptible to hydraulically irreversible fouling [10,11], and thus, they demonstrate smaller flux decline than polymeric membranes. However, fouling is unavoidable also in ceramic membranes, where pore clogging can occur both at the top layer and within the pores [12,13]. In turn, this leads to a significant increase in the hydraulic resistance and decrease of flux in the systems with constant pressure, or in the increase in transmembrane pressure (TMP) in constant flux systems [14].

Currently, one of the main challenges in the fully efficient application of ceramic membranes is the need to reduce membrane fouling and inevitable cleaning of the fouled membranes. The latter is critical not only to reduce fouling but also to retain membrane efficiency. In membrane cleaning, fouling is usually divided into reversible and irreversible. Reversible fouling reduces productivity and increases operational cost, while irreversible fouling increases operational complexity as well as reduces membrane lifespan [15]. These factors are of high importance in industries processing organic liquids with high fouling potential and thus cannot be avoided. In recent years, ceramic membranes have become more affordable, especially considering their longer lifespan [16,17]. Despite the high amount of information present for the mitigation of fouling and cleaning of different types of membranes, concise technical knowledge about the cleaning of ceramic membranes from organic foulants (such as proteins, polymers, colloids, and emulsions within the 2–100 nm range) is limited. This review summarizes available methods for non-reagent ceramic membrane cleaning as well as cleaning-in-place (CIP), their potential usability, limitations, and benefits as well as effectiveness. Hydraulically removable (non-reagent cleaning involved) fouling is interpreted as reversible fouling.

## 2. Fouling

**Figure 1:**
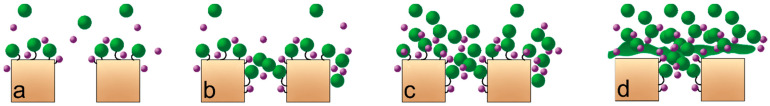
Membrane fouling progression: no changes in flux (**a**), permeate flux is affected (**b**), hydraulic resistance increased (**c**), and cake layer formation (**d**).

Generally, membrane fouling during the filtration process may be caused by various mechanisms (Figure 1) following each other or occurring simultaneously: adsorption takes place when membrane material interacts with particles present in the solution; this way, a thin layer of particles is attracted to the membrane without any flux (Figure 1a). Particles present in the solution can further promote partial or complete pore blockage, affecting permeate flux (Figure 1b). At the next stage, the deposition of particles occurs at the membrane surface, increasing hydraulic resistance (Figure 1c). The last stage is the formation of a cake layer (Figure 1d) at the membrane surface due to the aggregation of colloids and macromolecules rejected by the membrane [7,13,18]. Usually, the foulants are divided into organic, inorganic, and biological [19]; however, in mixed substrates (i.e., wastewaters), inorganic, organic, and biological fouling occurs simultaneously [20]. Inorganic fouling or scaling is caused by the deposition of salts and inorganic elements (i.e., Ca, Mg, Al, Fe, etc.) on the membrane surface. This is typically observed for reverse osmosis and nanofiltration membranes [21].

The adherence of hydrophilic and hydrophobic organic substances, generally proteins and polysaccharides, on the membrane surface is referred as organic fouling [19,22]. Higher polysaccharide concentrations in filtering solutions are linked to higher membrane fouling rates [23,24]. In water filtration systems, natural organic matter is considered the main foulant [12]. A part of wastewater associated with so-called effluent organic matter (EfOM) may also be of biological origin (proteins and polysaccharides) [25].

Biological fouling is linked to bacterial growth, metabolism, and deposition both in a bulk and membrane surface [19,21]. Biofouling usually starts with the deposition of individual cells or bacterial agglomerates on the membrane surface. Permeate flux going through the membrane provides attached cells with nutrients and dissolved oxygen [26], promoting cell multiplication, extracellular polymeric substances (EPS) formation, and soluble microbial product (SMP) secretion. In addition, even if 99.9% of the cells are removed from the surface, the rest will continue to grow, feeding on a substrate present in the filtering solution [27]. After all the aforementioned processes take place, the formed biofilm becomes more resistant toward hydraulic stress, chemicals, and antibiotics, making biofouling the most severe type of membrane fouling [13,28].

## 3. Reversible Fouling Treatment (Physical Methods)

Reversible fouling, leading to the reduction of the membrane permeation rate to 10–30% starts within several minutes of membrane operation, irrespective of thorough membrane selection for the specific liquid to be filtered [7,29]. Inevitably, reversible fouling is affected by membrane material. Significant fouling has been observed in alumina and zirconia membranes, followed by TiO_2_ and then SiC membranes [10]. Thus, focus on the cleaning of reversible fouling is essential. Furthermore, to postpone subsequent irreversible fouling, precautions must be taken in the reversible fouling stage. Generally, cleaning involves a change in the system hydrodynamics to remove foulants from surfaces using kinetic energy. Alternatively, membrane relaxation involving a membrane “rest“ period can be used to promote the diffusion of the foulant layer through a concentration gradient [30]. Backwash, backpulse, air bubbles, ultrasound [31], and other hydraulic techniques may be implemented to minimize reversible fouling. However, their efficiency can be affected by various factors (Table 1). 

### 3.1. Flushing/Rinsing

Hydraulic flushing detaches foulant particles through the turbulence of cross-flow toward the retentate side [32]. Bulk flow can be directed the same way as the feed stream or the opposite. Forward flushing (parallel to the membrane surface) can be used to remove the cake layer. The system configuration (amount of Tee sections, dead ends, etc.) is of great importance for flushing to be efficient [45]. Flushing is usually used in production industries before and after chemical cleaning to remove products from bulk and minimize the amount of chemicals.

### 3.2. Backwashing/Backflushing

Backwashing, as the name suggests, is performed by reversing the flow. Hydraulic backflush loosens the external side cake layer and removes foulants from membranes utilizing turbulent cross-flow [46]. The effectiveness of the backwashing depends on the duration and the frequency of the operations [47]. Flow rates for the backflushing of membranes vary from 10 to 400% of filtration flow rates [16]. To ensure turbulent conditions in ceramic membranes, backwashing is performed with flux at least two times higher than the regular flux through the membrane [41,46].

It has been indicated that a composition of a backwash solution is of big importance [31,41,43] for the effective removal of the fouling layer. In ultrafiltration membrane cleaning with backwashing, distilled water rather than permeate demonstrated better results [41,43]. In addition, the presence of monovalent cations and organic compounds promoted cleaning, whereas the presence of divalent cations (Ca^2+^, Mg^2+^) decreased cleaning efficiency [43].

Usually, hydraulic cleaning methods are used to prolong membrane cycles between chemical cleaning. However, hydraulic cleaning (backwashing) was reported to account for 17% of the total energy consumption for river water treatment or 0.425 kWh/m^3^ [48]. In addition, to implement backwashing, the filtration system has to be stopped, thus interrupting system operation [31]. For tubular membranes, an additional drawback is the difficulty of assuring appropriate and constant permeate flow rates throughout all channels of the multi-channel membranes [42]. It has been reported that backwashing may promote membrane fouling in hybrid (coagulation + ultrafiltrationmembrane) treatment systems [49] due to disturbing large flocs formed in reaction with coagulant and loosening the cake layer on the membrane surface.

### 3.3. Backpulsing

Backpulsing is superior to backwashing as it requires no system interruption [50]. Back pulses are usually short (less than 1 s) reverse flow pulses of permeate [50]. Increasing the backpulse amplitude allows performing effective cleaning in a short time [29]. Interactions of main parameters: amplitude × duration and amplitude × duration × frequency affect the final flux [51]. Backpulsing can be especially useful for enhancing protein transmission through the membrane for recovery [29,52].

### 3.4. Air Enhanced Backflushing

Air can be added to enhance the effect of turbulent flushing [46]. It is common to use air enhanced backflushing in short pulses (i.e., every several minutes) during normal membrane operation to increase the flux [53] and time between chemical cleaning cycles. However, air backflushing is not a suitable method for ultrafiltration membranes, as generated air bubbles are about two orders of magnitude bigger than membrane pore size [30]. Air flushing usually takes place before regular backflushing to detach debris from the surfaces [54].

### 3.5. Ultrasound

The use of ultrasound for cleaning membranes is a relatively new technology, but it is of high interest for membrane fouling mitigation and membrane cleaning on both a small and industrial scale. Ultrasound waves spread through a liquid medium via an alternating adiabatic compression and decompression (rarefaction) cycle waves, creating high and low-pressure oscillating regions [31,55]. In the rarefaction phase, negative net pressure is generated in the medium, and cavitation is triggered [31,32,55]. Cavitation bubbles collapse, leading to the formation of localized high temperatures (up to 5000 K) and pressures (up to 1000 atm) [32]. These extreme conditions result in high velocity (100–200 m/s) fluid movement [32], overcome the foulant–membrane interactions [29], and degrade foulant layers [35]. It was found that ultrasound increased the mass transfer coefficient of fluid across a membrane; however, ultrasound was not able to clean the inside of the membrane pores in ceramic membranes [36,56]. In general, using ultrasound for fouled membrane surface cleaning is reported to be effective for both flat sheet and tubular [36] ceramic membranes as well as polymeric membranes [29].

### 3.6. Electric Field

Application of the electric field is effective against the formation of a cake layer on the membrane surface by affecting electrical iterations between foulants and membrane material. An electric field is usually applied crosswise to the membrane employing electrodes on both sides of the membrane. The electric field can also be applied between the membrane and other electrode, or in the case of a ceramic membrane, it may serve as an electrode itself [40,57]. Membrane flux is directly proportional to the applied electrical field strength [38,40,58]. However, it is not reasonable to increase field strength above the critical strength, as it leads to increased power consumption and no additional benefits to the process. The critical field strength can be calculated:

(1) $$E_{critical}=\frac{J}{u_{p}}$$

where *J* is solvent flux at a given transmembrane pressure, and *u_p_* is the electrophoretic mobility of the particles. To lower process-related costs, a pulsing electric field can be implemented, meaning that the electrical field is applied in intervals. 

In the wastewater treatment process, a microbial fuel cell can be used as a green energy source for the generation of energy and fouling mitigation [59,60,61].

## 4. Irreversible Fouling Treatment (Chemical Methods)

Membrane operation and anti-fouling strategies may include pre-treatment (feed acidification, chlorination) and physical (hydraulic) cleaning. However, these methods are suitable for fouling mitigation rather than complete removal. Chemical cleaning or so-called cleaning-in-place (CIP) is the most commonly used method for the recovery of membrane flux [21].

**Figure 2:**
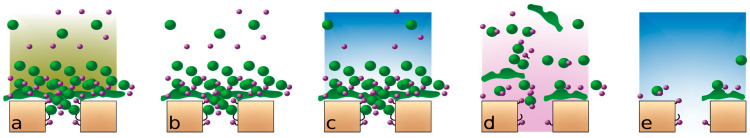
Typical membrane cleaning-in-place (CIP) procedure sequence including cake layer before treatment (**a**), draining (**b**) rinsing (**c**), chemical cleaning (**d**), and final rinsing (**e**).

It is assumed that cleaning costs represent 5–20% of operational costs [62]. In the food and beverage industry, about 20% of the time is spent on cleaning the equipment [63]; also, cleaning requires large quantities of water. It is considered that membrane lifetime depends on the cleaning cycles rather than the operation time [64]. Thus, it is important to make cleaning procedures effective and relatively cheap. In the food industry “cleaning out of place” (the system is disassembled prior to use) is also practiced [65]. Nevertheless, large-scale cleaning should be performed in an online regimen rather than stopping the production and taking membrane units off. For these reasons, simple and easily accessible chemicals should be used. A typical CIP procedure (Figure 2) may include an alternation of reagents and rinse cycles.

### 4.1. Cleaning-In-Place Procedures 

During CIP, the fouled equipment is cleaned online without dismantling the system. Effective cleaning depends on flow (kinetic energy), reagent concentration (chemical energy), temperature (thermal energy), and contact time. CIP procedures for ultrafiltration membrane systems are used to remove fouling caused by inorganic, organic, or biological matter. When CIP is performed in a single pass mode, it is referred to as single-use cleaning. This technique is usually performed for heavily fouled systems, such as ultra-high temperature processing food systems. Alternatively, the cleaning solution is recycled (recovery cleaning). Both approaches have their advantages and disadvantages. In single-use cleaning, the cleaning solution is always fresh, and the cleaning process should take less time. However, larger quantities of chemicals are required, and a higher environmental load is produced. Fewer chemicals and less energy are required to perform recovery cleaning. At the same time, more equipment (namely, recirculation pumps) is involved; thus, it needs higher investment and processing costs. Despite the selected method, initial flushing with water or deionized water should be performed to discard product residue from bulk, remove easily dissolvable substances (i.e., sugars and salts), and minimize active cleaning reagent consumption. Initial rinse is usually performed at the same temperature as all cleaning processes to avoid the densening of deposits and usually lasts 5–20 min [21]. An initial rinse at elevated temperatures (40–60 °C) may be performed for fat melting. Nevertheless, water rinse is required between different reagents (i.e., detergents, alkalis, and acids) to remove dissolved foulants and avoid the chemical interaction of reagents. However, the mild acidic rinse may be used after alkaline chemicals to neutralize high pH.

Temperature affects diffusion, mass transfer, and fluid characteristics [21]. The CIP temperature should not affect the cleaning process in a negative manner. In the food industry, it is considered that the best temperature for cleaning should be the same as the processing temperature [65]. This way, the fouling layer crosslinking and denaturation is avoided. In addition, the heating of liquids is a highly energy-consuming process. Every 1 °C CIP temperature decrease leads to a 1/60 decrease in energy consumption for fluid heating [63]. An energy analysis during membrane cleaning showed that a thermal process rather than fluid pumping accounts for the majority of the energy consumption [66].

The membrane cleaning schedule depends on the intensity of fouling and the types of foulants. Membranes involved in food production have to be cleaned daily unlike to desalination membranes and ones used for technological processes [67]. However, each case requires individual evaluation to mitigate intensive membrane fouling, save reagents, minimize the effect on the environment, and preserve membrane integrity and lifespan.

### 4.2. Cleaning-In-Place Reagents

Selection of the right reagent for membrane cleaning is crucial, and it mostly depends on the foulant. Typical reagents used for membrane cleaning and their cleaning mechanisms are summarized in Table 2 and Table 3, and discussed below.

#### 4.2.1. Alkalis

In larger-scale systems, it is very common to use strong alkaline reagents first (Table 3), as they effectively dissolve organic foulants such as fats, sugars, and proteins [65]. Alkaline reagents can also remove EPS and polysaccharides from the membrane surface [3]. Caustic induces saponification reaction with fats and oils, resulting in the formation of soap micelles [21]. Other important mechanisms of alkali lay in the alteration of liquid zeta potential and affecting electrostatic reactions that hold natural organic matter to the membrane surface [21]. Sodium hydroxide (NaOH) is preferred over potassium hydroxide (KOH), as NaOH has better solubility, higher cleaning efficiency, and a lower price [74].

Typically, NaOH is used in concentrations of 0.5–2 wt % [75]. However, for some applications, higher concentrations may be needed. The maximum permitted concentration should be checked, as excessive NaOH concentrations tend to induce crosslinking proteins, making them more rigid [65,76]. Ref. [65] measured total organic carbon (TOC) concentration by trying different NaOH concentrations as an effective way on how to determine an optimal NaOH concentration for CIP. Higher TOC concentration in cleaning solution indicates higher cleaning efficiency. NaOH initiates the hydrolysis process by increasing the solubility of different solutes [77], resulting in the loosening and dispersing of the cake layer [3]. NaOH dissolves proteins by alkali cutting the crosslinking that holds the proteins together [65]. In addition, it can be used in combination with surfactants to enhance the loosening effect and oxidants for effective bactericidal properties [78]. Ref. [3] used a combination of NaOH and sodium hypochlorite (NaOCl) to promote the removal of foulants from the metal sediments surface. 

#### 4.2.2. Acids

Alkaline cleaning is usually followed by acid cleaning. Acids dissolve inorganic sediments such as metal dioxides [21] and encrusted proteins [75]. Most often, nitric (HNO_3_) and phosphoric (H_3_PO_4_) acids are used. HNO_3_ is popular in the food industry to dissolve calcium precipitates [30]. Typical concentrations for HNO_3_ are 0.5–1.5 wt %. At higher concentrations, HNO_3_ may induce corrosion and affect polymer materials [65]. To avoid the negative effects of strong acids, weak acids can be used instead. In the food industry, acid is used due to its bacteriostatic properties [75]. The removal of divalent cations using acids or chelating agents eases the cleaning of membranes fouled by organic matter [21].

#### 4.2.3. Chelating Agents

Chelating agents aid to remove colloidal, chemical, and biological material as well as sulfate scales [21]. The most common chelating agents are ethylenediaminetetraacetic acid (EDTA), nitrilotriacetate (NTA), methylglycin diacetate (MGDA), phosphates, phosphonates (DTPMP, ATMP), polyphosphates, iminodisuccinate (IDS), and enzymatic detergents. EDTA is commonly used in dairy production to remove scales and prevent calcium and magnesium scaling [74]. However, EDTA forms stable water-soluble complexes, which means that heavy metals remain in water rather than undergo treatment in the biological WWTPs sludge. Later, they are discharged into the environment [74].

**Table 3:** Cleaning-in-place (CIP) procedures for ceramic ultrafiltration membranes. RT—room temperature, n/a—not available.

Membrane, Material, Configuration	Reagent	Concentration	Temperature, °C	Time, min	Flux Decline	CIP Regularity	Efficiency	Filtering Solution	Reference
Ultrafiltration (UF) Ceramic (α-Al_2_O_3_)	NaOH Ultrasil P3-14, Ultrasil P3-10	1% w/w n/a	RT	30–60			n/a	Galactosyl-oligosaccharides	[78]
UF and MF Ceramic	NaOH Water Citric acid	6% - 6%	RT 25 RT	30 30 30			n/a	Mains water	[79]
Microfiltration Ceramic (α-Al_2_O_3_)	Water NaOH Water Citric acid Water	- 2% - 2% -	RT 70-80 RT 70-80 RT	10 20 10 20 10			n/a	API effluent	[80]
UF Ceramic (α-Al_2_O_3_)	NaOCl H_2_O_2_	200 ppm (0.02%) 500 ppm (0.05%)	NM	15		Every 1.5 h	n/a	Lake water with electrocoagulation pre-treatment	[81]
UF Ceramic (α-Al_2_O_3_)	HNO_3_ NaOH	2% 2%	40 40	40 40			n/a	Sugarcane juice	[41]
UF Ceramic (α-Al_2_O_3_)	NaOH Free chlorine	1% 3000 ppm	40 40	60			n/a	Sugarcane juice	[41]
UF Ceramic Zirconite	Mix of NaOH and NaClO (w/w) water HNO_3_	1% 0.5% 0.5%	60	120 15			97.5%	Sugarcane juice	[3]
UF Ceramic Zirconite	Mix of NaOH and NaClO	1% 0.5%	60	120			79.8%	Sugarcane juice	[3]
UF Ceramic Zirconite	NaOH NaOCl	1% 0.5%	60	120 120			82.6%	Sugarcane juice	[3]
UF Ceramic Zirconite	NaOCl NaOH	0.5% 1%	60	120 120			80.5%	Sugarcane juice	[3]
UF α-Alumina Support Coated with LaPO_4_ nanofibrils	Mix of NaOH and NaClO HNO_3_	1% 0.5% 0.5%	n/a	180 30	98.65%	Every 60 min	87%	Sugarcane juice	[82]
α-Al_2_O_3_ 200 nm	2% (w/w) NaOH and 0.15 M HNO_3_		40	n/a	n/a		n/a	Rice wine	[83]
Al_2_O_3_ 10 nm	Hexane			~80 min	Reaching the mass concentration factor equal to 3.2		~100%	Crude soybean oil and hexane mixture, 32% in soybean oil	[84]
Ceramic 50 nm	Free chlorine HClO_3_ NaOH HNO_3_	250 ppm 20% 10%	n/a	20 - -	When flux dropped below effluent production rate required to match the influent flow		n/a	Simulated newsprint mill wastewater	[85]
Ceramic UF with Zirconite	Mixture of NaOH and SDS HNO_3_ Mixture of NaClO and NaOH	20 g/L 2 g/L 0.5% 250 ppm Cl_2_ 0.5 μg/L	50 50 30	30 30 15			~100%	Wastewaters from fish processing	[6]
ZrO_2_, Al_2_O_3_, TiO_2_ 100 nm	Citric acid NaOCl	1% 3000 ppm	n/a n/a	120 120			n/a	Surface (lake) water	[10]
ZrO_2_	Enzyme Maxatase®	5.0 /L	50	20			~100%	Whey protein	[86]
Aluminum Oxide 50 nm	Water jet	High pH bath NaClO (200 ppm free chlorine) adjusted to a pH of 11 Low pH bath distilled water adjusted to a pH of 2 using HNO_3_		60 25 min	TMP incrased to greater than 0.5 bar	Every 48 h		Real primary effluent wastewater	[87]

#### 4.2.4. Surfactants/Detergents

Surfactants are non-ionic chemicals that have both hydrophobic and hydrophilic properties. The key mechanism is the formation of micelles with fats, oils, and proteins [21]. Detergents help remove heavy biological and organic matter [21]. Formulated detergents have certain agents added to increase cleaning effectiveness. The main component of all formulated detergents is always an alkali or an acid. Additional components can include the following: (1) surfactants or wetting agents that lower surface tension, enabling them to wet a surface more effectively and make cleaning more efficient [74]; (2) calcium and magnesium ions sequestering agents to soften the water; (3) complex-forming agents that can only bind one metal ion per molecule in contrast to sequestering agents, which can bind to several metal ions; (4) oxidation agents that can boost cleaning effects. Examples are NaOCl and hydrogen peroxide [65].

#### 4.2.5. Disinfectants/Oxidising Agents

NaOCl is a common disinfection agent as it interferes with cell metabolism causing oxidative stress as well as lipid and fatty acid degradation [88]. In addition, being a strong oxidant NaOCl increases the hydrophilicity of organic molecules, promoting their detachment from the membrane surface [6]. Both NaOCl and H_2_O_2_ promote fouled membrane gel layer decomposition and detachment [3]. Strong inorganic acids, such as HCl, H_2_SO_4_, and HNO_3_ affect the solubility of metals [77], making them effective for the removal of metal salts. Metal oxides are small in comparison to organic molecules and tend to adsorb within membrane pores or in the immediate vicinity of the membrane surface, whereas organic molecules will adsorb on top of metal sediments. For this reason, a combination or alternation of reagents is often used for CIP. Controversial information is available concerning this topic (Table 3). [79] tested alkali–acid and acid–alkali sequenced cleaning procedures for ceramic membranes and found no significant difference in results.

#### 4.2.6. Enzymes

Enzymes are highly effective and selective catalysts aiming for a specific target. For this reason, the selection of a proper enzyme depending on the presented foulants is crucial. Enzymes demonstrate the best performance when the isoelectric point of the cleaning solution corresponds to that of the enzyme [64]. Enzymes actively break down proteins at bacterial attachment sites, making them effective for biofilm removal [86]. In a study for the cleaning of ceramic ultrafiltration membranes used for whey protein fractionation, enzymes showed high efficiency (close to 100%) in a short time (20 min) [86]. The cleaning effectiveness depends on the time, pH of the solution, temperature, and concentration of the enzyme. The dose of the enzyme should be carefully evaluated not only due to the high cost of enzymes but also because higher than necessary concentration on an enzyme may decrease the cleaning efficiency [86]. This phenomenon may be attributed to secondary membrane fouling caused by the enzyme itself or by the redeposition of solutes on the membrane surface [73,86,89]. Advantages in the utilization of enzymes are usually the relatively low (from 0.01%) concentrations needed [90] and the possibility to reuse the cleaning solution [86]. However, the enzymatic activity decreases by approximately 30% with every cycle [86], and the reagents are expensive when compared to other chemicals. Furthermore, it was reported that after 16 enzymatic cleaning solution reuse cycles, the fouling rate of the membranes used for wastewater treatment increased four times [90].

## 5. Future Perspectives

In large-scale systems, CIP procedures are robust and include strong chemicals to achieve the necessary effectiveness and optimize costs. While cleaning strategies are being tailored to meet systems’ hygiene requirements, environmental impact is unavoidable with the chemical cleaning methods being used. New environment-friendly reagents (i.e., enzymes and enzyme-based solutions) are gaining more popularity, although the costs of these chemicals are high for industrial-scale applications at the moment.

The use of anti-fouling or self-cleaning membranes is another promising perspective. In the field of fouling mitigation strategies, the development of new coatings and coating technologies allowing the production of neutral or negatively charged membranes has great potential. The electrostatically neutral membranes generate no electrical charge, thus minimizing the attraction of foulants. Negatively charged membranes repel foulants by electrostatic repulsion force. As a result, lower flux decline, lower membrane fouling, and higher flux recovery is achieved.

The emerging technologies for the development of self-cleaning membranes are of big interest. Foulants are being removed from the membrane surface with the use of an electrically conductive layer. Hydrogen bubbles are generated by electrochemical redox reactions at the membrane surface. These bubbles disturb the foulant layer and promote high flux recovery.

## 6. Summary

Ceramic membrane fouling mitigation strategies and CIP procedures consume a great amount of energy and have an impact on the environment and system as such. For these reasons, a careful selection of CIP procedures should be performed. Firstly, the understanding of the feed composition and identification of foulants is necessary. Different kinds of foulants ask for different cleaning strategies. High amounts of reagents may lead to adverse effects, generate high expenses, and contribute to the chemical load of wastewater treatment systems and the environment. Hydraulic conditions should be analyzed and optimized. Higher flux and pressure can affect the density of the foulant layer and enhance the concentration polarization on the membrane surface. 

To minimize the irreversible fouling of ceramic membranes affected by organic fouling, precautions must be held in a reversible fouling state, implementing an effective hydraulic cleaning strategy. From all, backpulsing is the most suitable technique, since it requires no system interruption and has proven to be effective in a short operation time. The duration (usually 1–3 s) and regularity of pulses should be evaluated individually for the given system. 

The long-term fouling and cleaning of ceramic membranes are usually difficult to scale up under laboratory conditions, so industrial setups rely on common methods and reagents practiced for many years. Recommended CIP procedures for the membranes fouled with organic material include an alternation of detergents (surface-active solutions or NaOH) and strong oxidants, e.g., active chlorine releasing reagents, to loosen the organic gel layer and then deep clean the membrane. The most common concentration of NaOH applied for organic foulants is 1%. However, it should be lowered to 0.3–0.5% when proteins are present in the filtering solution. It should be noted that the use of harsh chemicals and temperatures up to 80 °C has not demonstrated any significant damage to ceramic membranes.

## Figures and Tables

**Table 1:** Factors affecting the efficiency of non-reagent membrane cleaning methods.

Method/ Membrane Configuration	Affecting Factors	Drawbacks
**Ultrasound** Flat sheet Tubular	**Ultrasound frequency.** Lower ultrasound frequencies make cleaning more efficient than higher frequencies [32,33,34,35].	Fail to provide a uniform distribution of the ultrasonic energy to the fouled membrane surface [29,36]. Damage to the ceramic membranes was observed when using high powers [37].
	**Ultrasound power intensity.** Sonochemical effects (amount of bubbles, hydrodynamic turbulence) boost with the increase of ultrasound power intensity [32,35].	
	**Temperature.** The best conditions for effective cavitation were reported at 60–70 °C. When the temperature was decreased to 40 °C or raised to 85 °C, the cavitation efficiency decreased by half [29].	
**Electric field** Flat sheetTubular	**Zeta potential of a feed.** **Electrical field strength.** The maximal efficiency (lowest fouling degree) is achieved when an electrical field strength is close to critical [38].	Intensive corrosion or expensive corrosion-resistant electrodes [39]. Potential risk of electrocoating a membrane in hard water [40].
**Backwashing** Flat sheet Hollow fibre Tubular	**Pressure.** For effective particle removal, backwash pressure has to be higher than the membrane operating pressure [29].	Intensive energy consumption [30,41]. Hard to ensure constant and uniform backflow through multichannel membranes [42].
	**Composition of backwash solution.** Backwashing is more effective using deionized water, rather than permeate [41,43].	
**Backpulsing** Flat Sheet Tubular	**Amplitude.** An increase in amplitude allows decreasing the cleaning time [29,44].	
	**Frequency.** The short duration of back pulses is key for effective foulant removal [44].	

**Table 2:** Chemical membrane cleaning reagents and their cleaning mechanisms.

Category	Mechanism	Common Chemicals	Reference
Alkalis	Dissolving organic and inorganic material, saponification of fats and oils, hydrolysis of proteins.	NaOH, KOH	[30,68,69]
Acids	Solubilization and chelation of metal oxides, dissolution of scales.	HCl, H_3_PO_4_, HNO_3_, citric acid.	[21,30,64]
Oxidants/Disinfectants	Disinfection, increase of hydrophilicity, oxidation of foulants.	NaOCl, free chlorine, H_2_O_2_, peroxyacetic acid	[31,70,71]
Chelating agents	Forming complexes with metals in order to keep them in solution.	Citric acid, ethylenediamine-tetraacetic acid	[27,62,70]
Surfactants	Increase surface wettability, lower surface tension, increase the solubility of foulants, emulsification, dispersion of foulants.	Surfactants, detergents (SDS, sodium dodecylsulfate)	[30,64,72]
Enzymes	Split or hydrolyze protein–peptide bonds, disintegrating the protein.	Protease, lipase, commercial enzyme mixes	[21,73]

## Data Availability

Not relevant.
